# Adolescent Girls and Young Women’s Experiences with Disclosing Oral PrEP or Dapivirine Vaginal Ring Use: a Multi-Country Qualitative Analysis

**DOI:** 10.1007/s10461-023-04109-w

**Published:** 2023-07-01

**Authors:** Alinda M. Young, Noah Mancuso, Millicent Atujuna, Siyanda Tenza, Miria Chitukuta, Doreen Kemigisha, Kenneth Ngure, Ariane van der Straten, Morgan Garcia, Danny Szydlo, Lydia Soto-Torres, Sarah T. Roberts

**Affiliations:** 1 Women’s Global Health Imperative, RTI International, 2150 Shattuck Avenue, Suite 800, Berkeley, CA 94704 USA; 2 University of North Carolina at Chapel Hill, Chapel Hill, NC USA; 3 Desmond Tutu HIV Centre, Cape Town, South Africa; 4 Wits Reproductive Health and HIV Institute (Wits RHI), University of the Witwatersrand, Johannesburg, South Africa; 5 University of Zimbabwe Clinical Trials Research Centre (UZ-CTRC), Harare, Zimbabwe; 6 Makerere University - Johns Hopkins University Research Collaboration, Kampala, Uganda; 7 School of Public Health, Jomo Kenyatta University of Agriculture and Technology, Nairobi, Kenya; 8 ASTRA Consulting, Kensington, CA and CAPS, Department of Medicine, University of California, San Francisco, San Francisco, USA; 9 FHI 360, Durham, NC USA; 10 Statistical Center for HIV/AIDS Research and Prevention, Fred Hutchinson Cancer Research Center, Seattle, WA USA; 11 National Institute of Allergy and Infectious Diseases, National Institutes of Health, Maryland, MD USA

**Keywords:** HIV/AIDS, Disclosure, Adolescent girls and young women (AGYW), Eastern and Southern Africa, Oral PrEP, Dapivirine Vaginal Ring

## Abstract

Effective use of oral pre-exposure prophylaxis (PrEP) has been low among adolescent girls and young women (AGYW) in Eastern and Southern Africa, partly due to stigma and opposition from key influencers. Understanding AGYW’s experiences of disclosure of different PrEP modalities to key influencers may inform strategies to motivate uptake and adherence. We analyzed qualitative in-depth interviews and focus group discussions data from 119 participants in the MTN-034/REACH (**R**eversing the **E**pidemic in **A**frica with **C**hoices in **H**IV Prevention) study of oral PrEP and the dapivirine vaginal ring (ring) to explore AGYW’s disclosure experiences. We found that AGYW disclosure experiences varied across influencers and product type. The ring was disclosed less often to most influencers, except partners, because it was discreet. Oral PrEP was disclosed more often, because pills were more common and to avoid HIV stigma given that oral PrEP resembled HIV treatment. Ultimately, disclosure typically led most key influencers to support product use through reminders and encouragement. While disclosure yielded positive support from influencers, further community awareness of both PrEP products is essential to reduce potential opposition and perceived stigma.

**Clinical Trial Number**: NCT03593655.

**Supplementary Information:**

The online version contains supplementary material available at 10.1007/s10461-023-04109-w.

## Introduction

Adolescent girls and young women (AGYW) in Eastern and Southern Africa are disproportionately impacted by HIV, accounting for more than a quarter of all new HIV diagnoses despite representing only 10% of the total population [1]. Moreover, AGYW have 2–7 times higher risk of HIV acquisition than their male counterparts [2–5]. Daily oral pre-exposure prophylaxis (PrEP) has been shown to be an effective HIV prevention method for heterosexual women and has been offered for AGYW in the region through national programs and demonstration projects since 2016 [6–9]. More recently, the dapivirine vaginal ring (ring) has also proven to be efficacious in preventing HIV acquisition in Eastern and Southern Africa [10, 11]. The ring is designed to be worn continuously for one month and replaced with a new ring thereafter. The World Health Organization (WHO) recently recommended the ring as a supplementary method to oral PrEP for HIV prevention among women, but both rely on adequate adherence to be effective [12].

For AGYW using oral PrEP, fear of disclosure to key influencers (e.g., family, friends, and partners) has been one of many documented challenges to oral PrEP adherence [13–15]. These fears stem primarily from sexual and HIV-related stigma and misinformation about product use [16–18]. AGYW have also reported fear of disclosure due to community norms around adolescent sexuality and healthcare worker concerns about promoting behavior that is culturally taboo, such as multiple sexual partners or sex before marriage [19]. Positive experiences with disclosure can be an empowering way to combat stigma and misinformation, and AGYW who have had positive disclosure experiences have been reported to also have higher oral PrEP adherence [17, 18, 20, 21].

Little research has been conducted on ring use among AGYW in Eastern and Southern Africa. Among adult women, positive disclosure experiences among peers, partners, and family were reported to positively influence product acceptability and adherence [22]. AGYW ring disclosure experiences may differ from their experiences with oral PrEP disclosure as the ring is a new product and may be more discreet than oral PrEP [23]. Ring disclosure experiences may also differ from those of adult women since AGYW face additional social barriers, such as stigma related to age and sexual activity [24, 25]. Moreover, AGYW might be more financially and emotionally dependent on family members and partners, which can impede their ability to disclose if they predict opposition [26]. Understanding AGYW’s disclosure experiences with the ring compared to oral PrEP may help inform strategies to motivate uptake and adherence to both oral PrEP and ring use in this population. This paper draws on data collected in the Microbicide Trials Network (MTN) 034/REACH (**R**eversing the **E**pidemic in **A**frica with **C**hoices in **H**IV Prevention) trial to better understand AGYW’s disclosure experiences of the ring and oral PrEP (henceforth referred to as pills) to key influencers.

## Methods

### Study Design

**Fig. 1:**
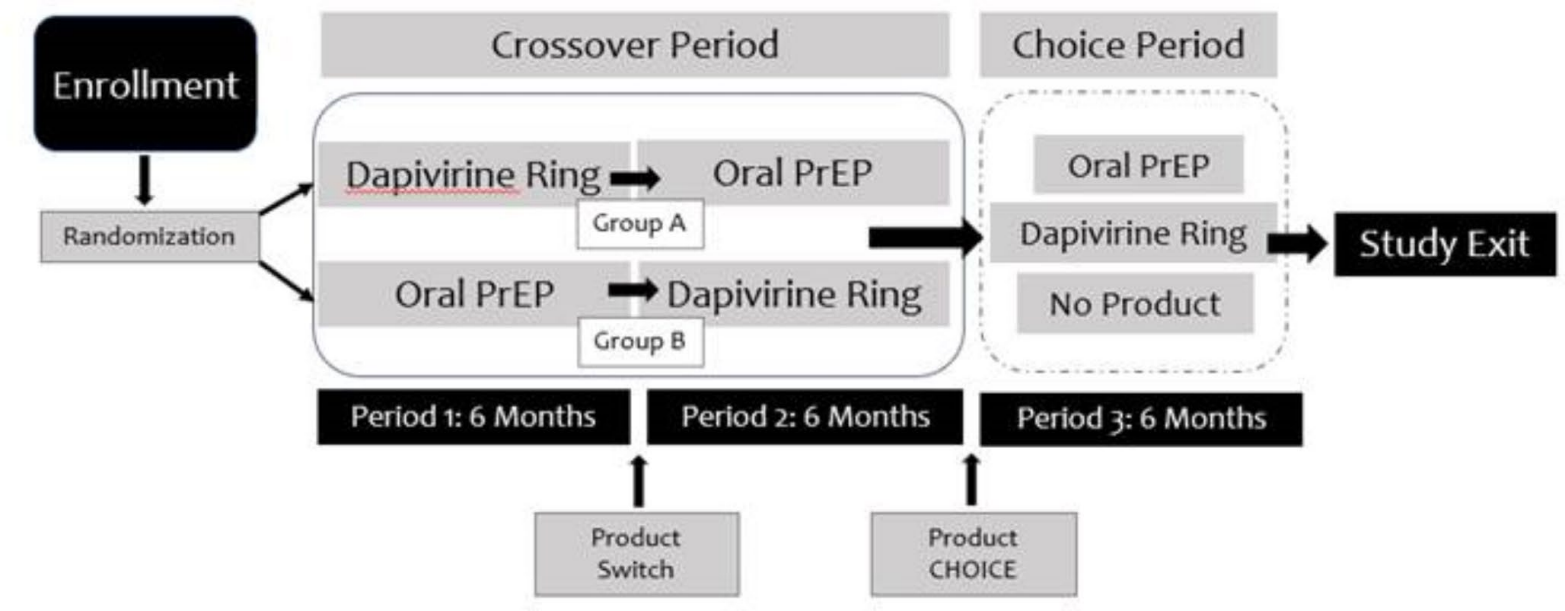
Study design

REACH was a phase 2a, randomized, open-label, crossover trial conducted at four research sites in three Eastern and Southern African countries: Johannesburg and Cape Town, South Africa; Kampala, Uganda; and Harare, Zimbabwe [27, 28]. The study aimed to collect safety and adherence data for the monthly ring and daily pills among AGYW and to understand individuals’ preference between the two products. The study enrolled 247 HIV-negative cis-gender^1^AGYW between the ages of 16–21 years old between January 2019 and September 2021. At enrollment, participants were randomized equally to receive the ring or pills for a period of six months (period 1) and switched to the other product for six months (period 2), referred to as the crossover period. In the third six-month period (period 3/“choice” period), participants were given a choice of pills, the ring, or neither product with an option to switch at any time (Fig. 1). All women were required to use a contraceptive method two months prior to study product use and throughout the study, however, they were allowed to discontinue contraceptive use if desired. More detailed information about the design, procedures, and primary findings have been previously reported elsewhere [27, 28].

### Procedures

All REACH participants completed Audio Computer-Assisted Self-Interview (ACASI) surveys at baseline and quarterly throughout the study. Each survey asked which or whether the participants’ primary sex partner, female family members, male family members, friends or other key influencers were aware of their product use.

In a nested qualitative component (N = 119), trained social scientists facilitated three sets of activities:

1. Sets of 3 serial in-depth interviews (n = 24 participants) (1 during each period after about 2 months of product use), averaging 60 min per IDI. Participants were randomly selected within strata defined by age group (16–17; 18–19; and 20–21) and period 1 product assignment.
2. 38 single in-depth interviews (IDIs), conducted at any time during follow-up, averaging 60 min each. Five single IDI participants also took part in FGDs later in the study. Candidates included participants who chose not to use a product in period 3 and those who experienced special circumstances such as seroconversion or violence. Those with special circumstances were nominated by site staff and approved by the qualitative management team, which was made up of individuals from the study’s Behavioral Research Working Group, Protocol Chairs, and the overall study management team.
3. 16 focus group discussions (FGDs; 4 per site with n = 62 total participants) during the choice period, averaging 90 min each. Participants who had used their chosen product for at least 2 months were purposively selected to ensure a mix of chosen products and adherence levels.

All discussions were conducted in the local languages and used semi-structured interview guides to explore disclosure of product use and subsequent reactions from key influencers, including family, partners, peers, and community. Audio recordings of the interviews were transcribed and translated into English at study sites. All transcripts were reviewed internally at the study sites before going through quality control by staff at the qualitative data management center.

### Analysis

From the ACASI data, we calculated descriptive statistics on product use disclosure for the subset of qualitative participants. For simplicity, we present the proportion of AGYW reporting any disclosure to each key influencer group during the crossover phase of the study (periods 1 and 2) when each product was first used.

For the qualitative data, a codebook was iteratively developed by members from the qualitative management team and research sites, through an inductive and deductive process. The codes were informed by codebooks from previous MTN studies, the protocol objectives, and two analytical frameworks (The Mensch Model and Psychological Empowerment Framework) [29, 30]. The frameworks were used to inform the design of the behavioral components of the overall study and the qualitative guides. Codes were iteratively refined through extensive team discussions and test application to several transcripts. Upon codebook finalization, all transcripts were coded using Dedoose software [31] by a team of six analysts from the qualitative data management center and one research site. Intercoder reliability was assessed using the Dedoose training tool to create tests using selected key codes and by a centralized transcript reviewer assigned on a weekly basis to review at least one coded transcript from each coder. Coding meetings were held weekly to ensure codes were reliably applied across the coders and to resolve any disagreements in code interpretation.

For this analysis, the lead author and a qualitative analyst reviewed the excerpts coded with the DISCLOSURE code divided into five main groups according to co-application of the Family, Partner, Peers, and Social Harms/Benefits codes, plus an “other” group that included a combination of the following codes: interpersonal, contextual factors, and organizational influencers (see supplementary Table 1). The lead author and analyst wrote summary memos for each code report. Code reports were only pulled for excerpts coded Peers external to the study and not co-coded with peers in the study. Co-authors from the four sites reviewed all memos along with the results outline and provided input on the interpretation of the results. In the analysis, we categorized product disclosure as voluntary, involuntary, or nondisclosure. Voluntary disclosure is defined as having disclosed all (full) or some (partial) relevant information pertaining to the study participation or product use. Involuntary disclosure is defined as participants being put in situations where they feel compelled or obliged to disclose all or some information about their study participation or product use against their wishes. Non-disclosure is defined as participants not providing any information about study participation or product use. Furthermore, we define key influencers (influencers) in this analysis as individuals (e.g., parents, friends, sexual partners) who hold significant influence over a participant’s social interactions, behaviors, and decisions. We looked to understand how these “influencers” impacted a participant’s view of the study and product use (e.g., uptake, support, discontinuation).

### Ethics Statement/Ethical Considerations

All participants provided written consent prior to participation and confirmed consent verbally prior to participating in qualitative activities. All quotes are labeled with pseudonyms to protect participants’ identities. The study protocol was approved by the Institutional Review Board or Ethics Committee at each site and was overseen by the regulatory infrastructure of the NIH Division of AIDS and the Microbicide Trials Network.

## Results

### Participant Characteristics (Table 1)

At study enrolment, among the 119 AGYW who took part in the qualitative component, 87% had completed secondary school and 22% reported earning income (Table 1). Over a third (37%) of participants had given birth at least once and 10% were either married or cohabitating. The majority (60%) reported living with their mother and 35% with their siblings. Only 19% reported living with their father. Most AGYW (87%) had a primary partner and of those, 61% reported relying on their partner for financial support. Moreover, 13% of the AGYW reported experiences of intimate partner violence from their partner within the last 6 months.

**Table 1:** Participant Demographics at Study Enrolment

	N	(%)
**Total**	119	(100)
Age - median (interquartile range)	18	(17–19)
Secondary education completed	104	(87)
Currently in school	47	(40)
Earns income	26	(22)
Parous	44	(37)
***Household members***		
Mother	71	(60)
Father	23	(19)
Siblings	42	(35)
Children	10	(8)
Grandparents	6	(5)
Husband/main partner/boyfriend	12	(10)
Other	15	(13)
Lives alone	2	(2)
***Primary Sex Partner***		
Has a primary sex partner	103	(87)
Partner provides financial support	63	(61)
***Violence***		
Intimate partner violence in past 6 months	15	(13)

*The percentages have been rounded off

### Prevalence of Product Use Disclosure (Table 2)

During the crossover phase, a large majority of participants reported disclosure of both products to at least one female family member (82%), at least one friend (75%), or partner (58%) but few disclosed both products to any male family member (22%). Additionally, among AGYW who only disclosed one product, the ring was slightly more often disclosed to partners than pills (14% versus 11%, respectively), while the pills were more often disclosed to at least one male family member than the ring (26% versus 8%, respectively). Moreover, 11% of AGYW reported non-disclosure of either product to their partners, and 40% to any male family members. Disclosure patterns were similar during the choice period.

**Table 2:** Characterizing who qualitative participants disclosed ring and/or pills use to during the crossover phase

	N	(%)
**Disclosed to**	119	(100)
***Both products***		
Primary sex partner	69	(58)
Any female family member	98	(82)
Any male family member	26	(22)
Any friend	89	(75)
***Only ring, not pills***		
Primary partner	17	(14)
Female family member	4	(3)
Male family member	10	(8)
Friend	8	(7)
***Only pills, not ring***		
Primary partner	13	(11)
Female family member	8	(7)
Male family member	31	(26)
Friend	7	(6)
***Neither ring nor pills***		
Primary partner	13	(11)
Female family member	6	(5)
Male family member	48	(40)
Friend	11	(9)

*footnote – participants were allowed to indicate “not applicable” and those who chose that option are not included in the table. *The percentages have been rounded off.*

### Qualitative Results Overview

Qualitative findings will be presented in the following sections: reasons to disclose or not disclose, including circumstances for involuntary disclosure and what information was disclosed, and reactions to disclosure, including immediate reactions, how participants dealt with opposition or garnered support, and overall disclosure outcomes. Because reactions to disclosure were similar regardless of whether it was voluntary or involuntary or which social influencer group was disclosed to, this section describes all reactions together.

### Reasons to Disclose or Not Disclose

Among REACH participants, reasons for disclosing or not disclosing product use or study participation varied depending on the product and the influencer. Participants decided to disclose to the influencers they perceived to be supportive. They reported disclosing the pills more often than the ring because they believed the pills would be associated with antiretroviral (ARVs) if people were unaware of the pills’ purpose or that pills were familiar to influencers. Others felt that non-disclosure was not an option as the pills could be easily seen when one was taking them, while other felt influencers were familiar with pills and therefore would not have negative reactions. As one participant expressed: “*They know about the pills because I was not going to be able to hide them at home while we stayed together.”* [Age 18, Cape Town, FGD] Additionally, two participants disclosed to their friends because they needed a place to store their pills since they had not disclosed to family members: “*Yaa, sometimes friends provide help…If I share a problem with them, they can advise me on what to do…For example, the issue of pills…I keep my pills at my friend’s home…I feared that I might have a challenge at home.”* [Age 18, Spilhaus, IDI] Some participants who chose to disclose ring use believed that influencers would be more receptive towards it than pills because it was a new product, that the ring would not be associated with ARVs, or in case of partners, that it could be felt during sex. Some participants felt that if partners felt the ring during sex, they would think participants are bewitching them, and therefore, disclosing ahead of time would eliminate this potential problem.

Some motivations to disclose were not product specific. Some participants disclosed to their friends so that they too, could benefit from participating in the study (including using the ring and pills and receiving counseling and routine health checks). Community-based study recruitment activities also meant that various key influencers were already aware of REACH and its purpose; so, participants did not need to provide details when they disclosed either product, making it easier to do so. Participants often found it easier to disclose products to their family members if families were familiar with HIV prevention or treatment and in rare cases, if some family members were involved in other HIV prevention studies. Other reasons for disclosure to family included family members being the ones to encourage participants to take part in REACH or at least one family member being aware of their participation because they signed the guardian consent for participants below 18. One participant shared that her aunt knew about her product use because she signed the consent form, but her mother did not: “*She [mother] is not easy, at least the other mother [aunt] of mine who brought me here knows about it.”* [Age 17, Kampala, IDI].

Common reasons for nondisclosure included fear of stigma, rumors, and discomfort related to having sexual health conversations. Participants also shared that disclosing study product use would lead to influencers assuming they were being “promiscuous” or involved in prostitution. One participant shared that if she disclosed her pill use, her mom would be upset and that “*She will ask me if I want to start dating the whole village.“* [Age 19, Kampala, FGD] Participants also expressed discomfort disclosing product use to family members because it would mean informing them that they were sexually active. Most participants generally avoided disclosing to male family members as they viewed HIV prevention and product use to be a female issue and further mentioned that disclosing would lead to uncomfortable questions from male figures: “*Just telling a father, it’s something heavy. He will think that ‘so you are having sex.’ That picture in mind that so this is what you are doing. I felt like it has that [effect] so that’s why I didn’t manage to tell him*.” [Age 17, Spilhaus, FGD] The pills in particular were not disclosed because of their similarities to ARVs and belief that they would be associated with having HIV, similar to what was previous stated above as being a reason for disclosing.

Many participants felt confident in using the ring without disclosing to key influencers, except for male partners, because they perceived the ring to be more discreet than pills. A few participants avoided disclosing as a way to prevent partners from thinking they were being bewitched by the ring. Others, on the other hand, spoke of waiting to disclose until their partners felt the ring during sex as early disclosure might lead partners to complaining that the ring was disturbing them. Furthermore, some participants feared disclosure of products to partners would create mistrust in the relationship or would lead partners to stop using condoms: *“I do not want to tell him because even if you tell him he will not agree. Even if you tell him that there is a drug that prevents HIV, they won’t agree they will say that you have HIV. When they see you on tablets, they will say that you have HIV, so for me I do not say it.”* [Age 18, Kampala, FGD].

Moreover, a couple of participants felt it was a personal choice to protect their lives and therefore, no one else needed to know or be involved. Other reasons for nondisclosure among “other” groups included avoiding inciting potential harm such as rape: “*The reason why I don’t want my community to know is because if something happens to me, since PrEP works to protect you from HIV, if I’m exposed, let’s say by someone who rapes me then I’m able to use it, so the fact that I don’t want people to know is that I think someone will come and…[take advantage].“* [Age 18, Johannesburg, FGD].

#### I. Circumstances of Involuntary Disclosure

Though rare, disclosure to key influencers was not always by choice because products were sometimes discovered inadvertently. Involuntary disclosure to family, friends, and “others” occurred most frequently with the pills when they were tangibly seen in bottles or while being taken. One participant described her involuntary disclosure to her mother: *“Yes, she [mother] asked me; ‘I always see you swallowing tablets, what is your health problem? The tablets you swallow are always in a tin!”* [Age 19, Kampala, IDI] Realizing the mother was worried about her health, the participant provided her mother the consent form and made sure to disclose her ring use when she had switched over. Involuntary disclosure to partners happened more often with the ring, when it was felt during sex: “*My experience with the ring is very good, but there were this one day, my embarrassing moment, I was with my boyfriend, so he felt the ring…When we were having sex, he felt the ring and took it out and asked, ‘what is this?’…”* [Age 19, Johannesburg, FGD] In rare cases, involuntary disclosure of study participation occurred to peers when participants were seen being picked up for their study visits by study staff or taking time off work to attend clinic visits. Participants in this sample decided it was better to disclose their study participation and product use instead of making up a different explanation.

#### II. What Information is Disclosed

Across influencers, many participants spoke of fully disclosing their study participation and the purpose of the study products. However, some participants reported partial disclosure, either by disclosing minimal information about the products but not their actual purpose, or by disclosing one product but not the other. As previously mentioned, pills were more often disclosed than the ring to all influencer groups except partners. Lastly, participants also based subsequent disclosure of the second product on how influencers reacted to their contraceptive use or first product use: *“I can’t tell him [about her pill use] he may refuse me so that I fail to participate yet I would like to complete my targeted time period and when I told him about family planning use, he wasn’t happy about it, he said “why are you using it?”* [Age 18, Kampala, IDI].

Other participants only disclosed their study participation, but not the actual purpose of the study or that they were using products: “*Even me, the person who knew was my mom and my grandmother and my uncles they only know that I am going to the clinic but to do what they don’t know.”* [Age 17, Johannesburg, FGD] Others gave alternative explanations about the study, including the clinic teaching girls’ crafts, education sessions to teach girls about how to protect their health, or a presidential initiative to assess their health status: “*I had not told my mum about the tablets… I just said to her that I’m in school… I then said, “It’s a school which invites girls once every month to teach them about health and self-protection.”* [Age 18, Spilhaus, IDI].

### Reactions to Disclosure

#### I. Immediate Reactions

Typically, influencers were happy that there were products to protect women from acquiring HIV. Influencers praised participants for taking the initiative to protect themselves. One participant described her supportive disclosure experience with her partner: “*So he [partner] said “okay, it is fine. I do not have a problem with it. You are preventing yourself, so I do not have a problem with you protecting yourself. There is no problem.”* [Age 20, Cape Town, IDI] Peers, in particular, appreciated that the products could protect the participants during situations of possible HIV exposure, for example, if the participants were engaging in sex while drunk, or did not know her partner’s HIV status. Peers also encouraged participants to use condoms in conjunction with study products as the products did not prevent other STIs.

However, some reactions were more negative, for the same reasons anticipated by those who chose not to disclose. In general, pills more often had negative reactions due to their common association with HIV. One participant said: “*They [cousin] were saying these pills are the same size as the one taken by the people who are HIV positive*.” [Age 21, Cape Town, FGD]. A minority of unsupportive influencers did not believe that daily oral pills and ring existed. Others expressed a general distrust in research claiming that participants were being used as “guinea pigs.” On a few occasions, influencers also believed participants were involved in satanism or that products would cause adverse effects later in life, such as infertility or cancer. Some partners immediately opposed participant’s use, as they either assumed that the products were contraceptives or that participants were being “promiscuous”. In these cases, product use amplified relationship issues that were already present prior to product use and in one rare occasion led to a participant being physically assaulted by her partner as he assumed the ring was the cause of her infertility. This participant, like many others, had not disclosed to their partner that to participate in the study, she was required to use a long-acting modern contraceptive (which was separate from the pills and ring).

Among peers, mainly classmates, negative reactions were often related to study procedures rather than the study products themselves. In particular, peers feared the blood draws and often thought participants were selling their blood. Lastly, there was a case where a participant started getting approached by men asking her whether she was giving out sex since she was now protected from acquiring HIV: “*… the male adults in the neighborhood they start approaching you like you are offering free sex coz ‘you are protected 100%’. People started approaching me that I never thought would approach me [saying] ’I heard that you are protecting yourself’ like I’m giving out free sex.”* [Age 18, Johannesburg, FGD].

#### II. How Participants Dealt with Initial Opposition or Garnered Support

To clear up misunderstanding from key influencers, some participants provided the consent form that explained the study and study products. Others called upon study staff to clear up misunderstandings. These approaches of involving study staff or using consent forms successfully garnered acceptance in most cases, even among partners that discovered the ring during sex. A participant, who is quoted earlier describing this type of involuntary disclosure, describes how she responded: “…*so I had to explain, give him information about what the ring is about it was just a long, long day. But as time went, I brought him the pamphlet from the study, he understood.”* [Age 19, Johannesburg, FGD] A couple of participants also spoke of having aunts or partners who explained the study products to other family members, in most cases mothers. There were also reports of supportive mothers stepping in to address misunderstandings pertaining to the products from partners or community members. However, participants also shared their frustrations in trying to explain the purpose of the study to their influencers: “*She [friend] said I’m doing it so that I become promiscuous that’s why I’m protecting myself. I told her no, it’s not all about that, it’s all about my safety, and she went on to say she’d never do it because it messes up your womb and you won’t be able to have children and causes changes in the body.”* [Age 17, Johannesburg, FGD].

#### III. Disclosure Outcomes

Ultimately, disclosure typically led key influencers to support product use through reminders and encouragement. Reminders were reported more frequently with the pills than the ring and included reminding participants to take the pills daily or not to remove the ring, sending WhatsApp messages to remind participants when it was time to take their medication, and sometimes giving reminders when it was time for their clinic visits. One participant from Zimbabwe spoke of her partner bringing her the pills if she had not taken them or had forgotten. In some cases, family members or partners provided transportation for participants. In others, family members timed their own medication to align with the participants’ as a kind of support system to remind each other. One participant shared her experience with receiving support from her peers: “*They were happy for me when I was taking, and they would support me and they would ask, ‘[Rose] have you taken your pills?”* [Age 17, Spilhaus, IDI].

Positive reactions from key influencers also allowed participants to share challenges (e.g., side effects) and strategize ways to cope with them. A mother, for example, encouraged a participant to buy pads and pantry liners to adjust to excessive vaginal discharge due to ring use. This participant shared, “*When I developed that rash, I also told her [mother], and she advised that I phone the staff here. And she is familiar to this [research experience] because she is participating in another study.”* [Age 17, Spilhaus, IDI]. Another participant shared how her partner encouraged her to adhere to the ring despite some side effects. She said, “*When I explained to him about the vaginal ring, he encouraged me to be strong and use it. Because, when I smelt the bad odor, I thought he hadn’t smelt it but later he told me …’there is some bad odor’ and I explained to him and he said that ‘Okay, since it is protecting your health then it is okay*.” [ Age 17, Kampala, IDI].

Participants who were criticized learned ways to adhere to their product despite opposition. Some participants described fighting back against partners who opposed their product use or their choice of products in period 3, and a few even opted to end their relationships: “*he got angry, and I told him that even if he gets angry, it’s for my own safety. He was angry for 3 months but eventually came back.”* [Age 21, Johannesburg, FGD] Others removed the ring before sex, or attended clinic visits while partners were away from home. Some ring users who had not disclosed also spoke of changing sex positions (e.g., not wanting to be touched in the vagina), while pill users either took their pills before visiting their partners, or away from their partner if they happened to reside together.

Additionally, some participants hid their pills at their mother’s or peers if they had not disclosed to family members or partners. Some participants were left feeling sad and unsupported upon disclosure: “*My friends as well, I told them about the REACH program and the only thing they could think of is “oh [Muncie]!” [many people outside of [Muncie] consider it as an unsafe place to go to] you know. It was very disheartening because it’s like okay I can support you in your stuff, but you can’t support something that will benefit you as well. It’s quite shocking and sad actually.”* [Age 19, Johannesburg, IDI] Others however, indicated they were not affected by negative reactions: “*I’m not concerned what the community was saying because if you want to know the truth come to me so I can explain these are not ARVs, but PrEP and PrEP is for this and that*.“ [Age 20, Johannesburg, IDI] In all cases, participants described persisting despite opposition; none reported stopping product use due to influencers’ attitudes or actions.

## Discussion

Findings from the quantitative data show that most AGYW did disclose to some people, especially to female family members and friends. The qualitative findings show that there are numerous individuals they did not disclose to due to various reasons. The reasons for disclosing were similar across all social influencer groups and were most often influenced by perception that influencers would be supportive and to head off misconceptions that might arise from involuntary disclosure. While some participants disclosed pills because of resemblance to ARVs and wanted the pills not to be mistaken for ARVs, others did not disclose for this very same reason. Influencers’ prior knowledge of HIV prevention or treatment methods and awareness of REACH or other clinical research in their communities also influenced whether participants disclosed. Similar to other studies, anticipated stigma, judgement, and rumors often deterred AGYWs from disclosing [17, 18, 32, 33]. While most reactions to product use disclosure were positive, in some cases, disclosure led to conflicts, even after participants provided additional information. For most participants, disclosure ultimately led influencers to support participants’ adherence through reminders and encouragement.

Study participants were comfortable using the ring without disclosure to all key influencers except for male partners, whom they feared would discover it during sex. The foreignness of the ring made partners make incorrect assumptions about its purpose, whereas some initially thought it was witchcraft, others worried that it was a contraceptive. With the pills, however, male partner opposition was related to misconception about HIV and AGYW having “multiple sexual partners”. A qualitative study of ring acceptability among AGYW in the United States also found similar outcomes (e.g., contraceptive, promiscuity, and HIV) to partner disclosure of ring use [34]. In REACH, a few women reported removing the ring before engaging in sex. While this tactic may avoid involuntary disclosure and thus might prevent potential social harms, it impedes the effectiveness of the ring [34–36]. Similar to REACH, a study on contraceptive ring use reported that some women delayed disclosure of their ring use to see whether their partners would feel it or disclosed to avoid relationship conflicts [36]. In a recent phase III trial among adult women in Eastern and Southern Africa using the vaginal ring, a good number of women reported opposition to ring use from male partners, and low ring acceptability to male partners was associated with nonadherence [37]. Findings from REACH indicate that because pills are harder to hide, participants wanted to disclose to a wider variety of people and may need more support and counseling on how to disclose. Participants were comfortable using the ring without disclosing to people, except to partners, and therefore, counseling strategies should emphasize partner-related disclosure concerns.

Despite many studies in these communities and program rollout, pills are still ‘hidden’ which can lead to opposition from influencers and adherence challenges. Participants in this study reported individuals in their inner circles having misconceptions about pills, with many assuming it is used for treatment. Other studies have reported similar findings [17, 38]. Some participants, however, often could convince them to eventually support product use by providing more information and education about the products. Community education is vital in changing the narrative about HIV prevention and treatment options by highlighting that even though both products contain similar drugs, their purposes are vastly different (e.g., treat vs. prevent). Similar to a previous study on pill disclosure among AGYW in South Africa and Zimbabwe, participants reported experiencing HIV stigma from various influencers, arising when the pills were mistaken for HIV treatment, and sexual stigma when the pills and ring were thought to promote sexual promiscuity [17, 18, 32]. Furthermore, while in prior studies, peers external to the study have been reported to hold negative reactions upon disclosure [39], REACH highlighted that peers can actually be good supporters to women who choose to use HIV prevention methods and can act as a support system for women that are unable to gain support elsewhere. Indeed, adequate education to this social group is essential, and future research should focus on engaging communities in the rollout of the ring and provide further education on pills, through multiple platforms such as social media outlets and higher learning institutions. Studies have reported that advertising pills or family planning methods in social media or in the community increased adherence and confidence that the products were safe and effective [17, 40].

Additionally, we recommend supporting women to explain the purpose of the ring and daily oral pills and garner support during disclosure to their influencers. In REACH, some key influencers seemed more receptive to explanations from study staff, considering them to be expert sources, rather than from the AGYW themselves. This has major implications to programmatic roll-out as such experts may not be available to counsel or educate influencers. Therefore, women should be equipped with information and counseled to comfortably and convincingly relay the information to key influencers. It also is essential that we identify more credible stakeholders in the community to spread or amplify communication messages about HIV prevention, and can be allies for women if needed, to lessen the burden on the users themselves. Furthermore, clinicians in the public and private sectors should be educated on these products in instances where influencers seek further information after users disclose. Prior studies have found that lack of awareness about emerging HIV prevention methods, like the ring, among public healthcare providers leads to barriers for women joining research studies or using the products [38]. Rumors that women are using experimental drugs have been seen to shake women’s confidence and trust in the research or their rationale for participating in the study while healthy [38]. And though it might be difficult to implement, countries should start looking at ways to incorporate these products in their sexual and reproductive health curriculums for both the providers and AGYW.

Our study presents some limitations. Our findings are limited to AGYW in the trial setting and therefore cannot be generalized to the broader population. Because all study sites were in urban locations, we miss the perspectives of those who reside in rural areas. Besides, study participants were recruited from areas around the clinic and are likely familiar with clinical research and the study teams. Because they agreed to participate in the study and to be interviewed, AGYW in this study may have viewed biomedicine more favorably than those who did not participate. Additionally, most AGYW had higher than primary level education, and therefore, their responses and experiences may differ from AGYW with lower education. Finally, our study did not explore disclosure to other key influencers such as chiefs and religious leaders, which is a gap that may need to be filled in future research. Religious leaders and other traditional key influencers have been reported in previous MTN studies as potential trusted source of influence when it comes to the use of biomedical HIV prevention technologies [41]. However, the study also had major strengths. It is the first study looking at ring use among 16–17-year-olds in Eastern and Southern Africa and includes AGYW from three Eastern and Southern African countries that are heavily affected by the HIV epidemic. The quantitative data and large qualitative sample, using IDIs and FGDs, allowed us to understand the extent of disclosure, AGYW’s experiences with disclosure of the two HIV prevention products, the context of disclosure, and how disclosure experiences differed among various influencers, which are key for introducing new products and encouraging uptake and continued use. Lastly, data collection during different periods of the study allowed participants to report their experiences with each product at the time of use rather than retrospectively, reducing recall bias.

## Conclusion

In conclusion, disclosure experiences differed depending on product and type of influencers. While many participants found it easy to disclose both products to most key influencers, others were selective in which product or to whom they disclosed. Pills were most often disclosed because influencers were familiar with its form and because participants wanted to avoid potential HIV stigma from association with HIV treatment. On the otherhand, the ring was mostly disclosed to partners, as participants feared it could be felt during sex, however, considered it more discreet than pills and therefore, not always necessary to disclose to other influencers. Our findings show that community education and awareness about the ring and pills is vital in improving product uptake and user experiences while at same time addressing HIV and sexual stigma. Most participants that disclosed their product use reported having better adherence as disclosure alleviated fears of product misconceptions from influencers. However, beyond the community education, AGYW should be provided with counseling and necessary skills to disclose product use when desired with confidence and fear of judgement. REACH showed that key influencers could act as positive enforcement to encourage product adherence through pill reminders and encouragement of consistent ring use. Partaking in these strategies will mitigate challenges to adherence among AGYW by reducing potential key influencer opposition and perceived stigma, as well as improving acceptance of these biomedical prevention products.

### Electronic Supplementary Material

Below is the link to the electronic supplementary material.

Supplementary Table 1

## Data Availability

The data that support the findings of this study are available from the corresponding author upon reasonable request.

## Footnotes

1 At screening, participants were assumed to have been assigned female sex at birth and were not asked to report their gender identity. However, required safety pelvic exams that occurred during the screening process would later confirm sex at birth.
