# Supplementary material for: Adolescent Girls and Young Women’s Experiences with Disclosing Oral PrEP or Dapivirine Vaginal Ring Use: a Multi-Country Qualitative Analysis
Source: AIDS Behav. Author manuscript; Available in PMC 2023 Dec 1. (PMC10598125; doi:10.1007/s10461-023-04109-w)
Supplement: Supplementary Material [file NIHMS1932398-supplement-Supplementary_Material.docx]

**SUPPLEMENTARY CONTENT**

**Table S-1: Codes used in this Analysis**

| **Codes** | **Definitions** |
| --- | --- |
| Disclosure | Discussion of whom participant did or did not tell about study participation and product use, including voluntary partial or full disclosure. Include discussions of involuntary or unintentional disclosure of product use or study participation (e.g., partner felt ring during sex). |
| Interpersonal | Comments about other influencers (e.g., neighbors). Use only if child codes are not applicable. Comments regarding study staff should be coded as STUDY. Code ORGANIZATIONAL INFLUENCERS if comments on religious leaders, teachers, employees, etc. |
| Family | Any discussion of family members, including their influence on participant’s view of the study or study products. |
| Partners | Discussion about sexual partner(s) including, number, type, primary partner/spouse, communication, decision-making, influence on participant's study participation or product use. Include discussions of past/ex-partners. |
| Peers | Any discussion of friend groups, including their influences on participant’s view of the study or study products. Also include virtual or social media friends (e.g., WhatsApp, Facebook, etc.). |
| Social Harms/Benefits | Use to capture described benefits OR harm related to study participation or product use. Double code with other appropriate codes, e.g., VIOLENCE, DEVELOPMENTAL GROWTH, MONEY. |
| Organizational influencers | Discussions of other organizations/institutions besides the study clinic (e.g., work, school, church. Include comments on teachers, employees, religious leaders, social workers, etc. |
| Contextual factors | Include discussion of the social, cultural, or structural context in which the participant is living. May include local practices, urban/rural location, HIV prevalence, community violence (including rape), sociocultural norms, or other discussions of the community. Include comments about community-level financial/food security (or insecurity), etc. Also include comments about the level of community knowledge about PrEP or the REACH Study, co-code with the product they know/don’t know about (e.g., PILL or RING) |
